# Common DNA Variants Accurately Rank an Individual of Extreme Height

**DOI:** 10.1155/2018/5121540

**Published:** 2018-09-04

**Authors:** Corinne E. Sexton, Mark T. W. Ebbert, Ryan H. Miller, Meganne Ferrel, Jo Ann T. Tschanz, Christopher D. Corcoran, Alzheimer's Disease Neuroimaging Initiative, Perry G. Ridge, John S. K. Kauwe

**Affiliations:** ^1^Department of Biology, Brigham Young University, Provo, UT 84602, USA; ^2^Department of Neuroscience, Mayo Clinic, Jacksonville, FL 32224, USA; ^3^Department of Oncological Sciences, University of Utah, Salt Lake City, UT 84112, USA; ^4^Department of Psychology, Utah State University, Logan, UT, USA; ^5^Center for Epidemiologic Studies, Utah State University, Logan, UT, USA; ^6^Department of Mathematics and Statistics, Utah State University, Logan, UT, USA; ^7^Alzheimer's Disease Neuroimaging Initiative, University of Southern California, Los Angeles, CA 90089, USA

## Abstract

Polygenic scores (or genetic risk scores) quantify the aggregate of small effects from many common genetic loci that have been associated with a trait through genome-wide association. Polygenic scores were first used successfully in schizophrenia and have since been applied to multiple phenotypes including multiple sclerosis, rheumatoid arthritis, and height. Because human height is an easily-measured and complex polygenic trait, polygenic height scores provide exciting insights into the predictability of aggregate common variant effect on the phenotype. Shawn Bradley is an extremely tall former professional basketball player from Brigham Young University and the National Basketball Association (NBA), measuring 2.29 meters (7′6^″^, 99.99999th percentile for height) tall, with no known medical conditions. Here, we present a case where a rare combination of common SNPs in one individual results in an extremely high polygenic height score that is correlated with an extreme phenotype. While polygenic scores are not clinically significant in the average case, our findings suggest that for extreme phenotypes, polygenic scores may be more successful for the prediction of individuals.

## 1. Introduction

Polygenic, or genetic risk, scores are aggregate measurements of the effects of multiple common genetic loci that are associated with a trait. First used in schizophrenia [1], they have been applied to many complex traits such as multiple sclerosis [2], rheumatoid arthritis [3], and cardiovascular risk [4]. However, polygenic scores are not generally expected to be clinical predictors of an individual's phenotype. For example, Machiela et al. observed that the calculated AUC for the prediction of breast cancer from the polygenic score did not exceed 53%, which suggests that more validated variants (increased sample size) are necessary for a better prediction or that other factors besides common variants account for a large part of the disease phenotype [5]. Similarly, Evans et al. found that while adding genome-wide variant information can slightly improve prediction accuracy, it is unlikely to be used for the prediction of individual phenotypes until larger datasets can improve the number of validated associated variants [6].

Most phenotypes (e.g., height, Alzheimer's disease, Parkinson's disease, etc.) are complex and polygenic, and our understanding of the underlying biology is limited because of high data dimensionality and small sample sizes. Approximately 80% of adult height variation has been attributed to genetic factors [7–10], and common SNPs are believed to account for approximately 50% of that variation [11, 12]. The Genetic Investigation of ANthropometric Traits (GIANT) consortium recently identified 697 SNPs across 423 loci that explain 20% of adult height heritability and further demonstrated that the 2000, 3700, and 9500 most significantly associated SNPs explained 21%, 24%, and 29% of height variation [10], respectively. Using 160 of these SNPs, which explain 10% of variation in height as reported by the GIANT consortium, Chan et al. observed that weighted polygenic allele scores were as predictive as expected in the extreme height phenotypes [13]. This conclusion was also validated by Liu et al., who reported an AUC of 0.75 for a weighted allele score prediction for 180 SNPs on tall stature [14].

**Figure 1:**
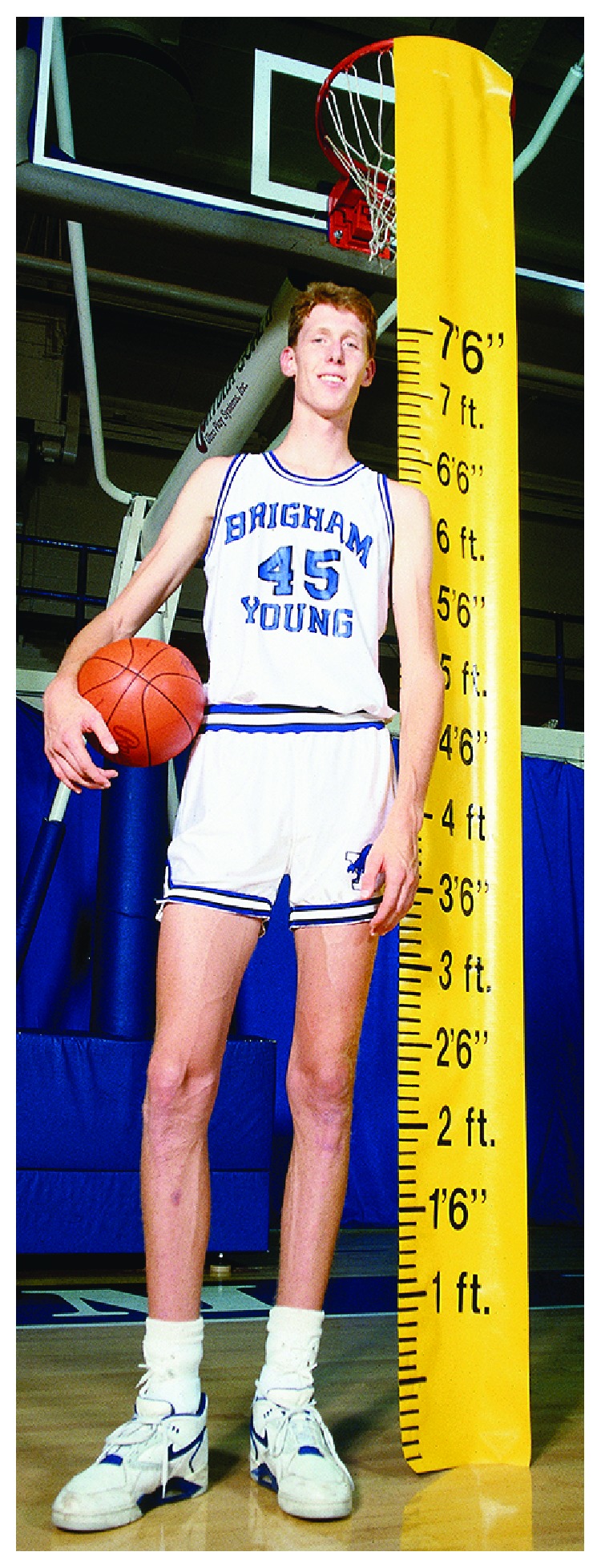
Shawn Bradley is 2.29 m (7′ 6^″^) tall with no known medical conditions. Mr. Bradley played basketball for Brigham Young University from 1990 to 1991. He played in the National Basketball Association from 1993–2005. Photo courtesy of BYU photography.

Shawn Bradley is an extremely tall former professional basketball player from Brigham Young University and the National Basketball Association (NBA), measuring 2.29 m (7′ 6^″^) tall (Figure 1) and has no known medical conditions. Mr. Bradley's height is 8.6 standard deviations (standard deviation = 6.05 cm) above the average height for US males (176.8 cm), putting him in the 99.99999th percentile [15]. While height is known to be polygenic, exceptional outliers for height and other phenotypes remain intriguing because their rarity may present exciting genetic insights. Possible explanations for their rare height may include a combination of rare genetic variants, environmental factors (e.g., diet) and an extremely rare combination of common SNPs. Here, we present evidence of a relationship between common SNPs and an extreme polygenic phenotype and demonstrate that in Mr. Bradley's specific case, the polygenic score predicts his height ranking as expected.

## 2. Materials and Methods

### 2.1. Sample Collection and Sequencing

The Cache County Study on Memory Health and Aging was initiated in 1994 [16] and consists of 5092 participants representing approximately 90% of the Cache County population aged 65 and older in 1994. Specific details about data collection, obtaining consent, and phenotyping individuals in the Cache County population were reported previously [16], and other additional information on this dataset can be found in previous reports [16, 17].

Whole genome sequences (WGS) from 809 individuals (432 males, 354 females, and 23 unknown) were obtained from the Alzheimer's Disease Neuroimaging Initiative (ADNI) database (http://adni.loni.usc.edu). ADNI is a large collaboration from several academic and private institutions, and subjects have been recruited from over 50 sites across the US and Canada. Currently, over 1500 adults (ages 55 to 90) participate, consisting of cognitively normal older individuals, people with early or late MCI, and people with early stage Alzheimer's disease. For up-to-date information, see http://www.adni-info.org.

We combined WGS from ADNI with WGS for 211 individuals (82 males and 129 females) from the Cache County study. All samples were sequenced using the Illumina HiSeq technology at an average of 30x coverage. We sequenced Mr. Bradley's exome using the Ion Torrent and the Ion Ampliseq Exome Kit at an average coverage of 30x. Sequence data from all studies were mapped to the human reference genome, version GrCh37 with BWA (Burrows-Wheeler Aligner) [18]. We further genotyped Mr. Bradley using the Illumina HumanOmniExpress chip and imputed additional SNPs using Impute2 [19] and the 1000G reference panel [20]. Subsequently, we filtered imputed SNPs with low information (info <0.4). Mr. Bradley and all individuals in the ADNI and Cache County cohorts are of Northern European ancestry [21].

SNP data from the Alzheimer's Disease Genetics Consortium (ADGC) were used to examine patterns of linkage disequilibrium. The ADGC consists of 32 studies collected over two phases that include 16,000 cases and 17,000 controls. All subjects were self-reported as being of European American ancestry. More information about this dataset can be found in the study of Naj et al. [22] and the ADGC data preparation description [23].

### 2.2. Analyses

The GIANT Consortium reported 22,539 genome-wide significant SNPs associated with human height. We extracted these SNPs from the ADGC data and identified unique tag SNPs within each LD block to (1) estimate the number of unique signals in the GIANT data and (2) prevent counting the same signal more than once. We identified tag SNPs using default settings in Haploview [24] for each chromosome individually (*r* ^2^ = 0.8). We then extracted as many of the remaining SNPs as possible from Mr. Bradley's data, the ADNI samples, and Cache County samples. We calculated an additive polygenic height score [25] for each individual and their respective ranks in the distribution of height scores. We also calculated the maximum possible score across the selected SNPs.

To estimate the number of SNPs needed to elevate Mr. Bradley's height score to the highest in the distribution, we performed a random selection of SNPs (bootstrap) at various SNP-set sizes ranging from 100 to 2000 SNPs, recalculating Mr. Bradley's height score and rank each time. We performed 1 million replicates for each SNP-set size and measured the range (minimum and maximum), first and third quartiles (25th and 75th percentiles), and the median for each SNP-set size.

We also explored the difference in height scores between the observed distribution of height scores amongst the 1020 individuals from ADNI and Cache County compared to the null distribution, assuming no evolutionary constraints. We simulated genotypes and height scores across the extracted common SNPs for 20 billion individuals. Specifically, for each SNP, we randomly chose one of three possible genotypes and calculated the simulated individual's height score.

Understanding whether Mr. Bradley's height is attributed to an increased proportion of heterozygous or homozygous genotypes associated with increased height could shed additional light on whether the SNP effects are additive or nonadditive (i.e., being homozygous has a greater effect than the sum). We tested for a difference between Mr. Bradley's genotype distribution and the average ADNI and Cache County genotype distribution using a goodness-of-fit test. Alleles with a positive effect size are associated with increased height, while alleles with a negative effect size are associated with decreased height. A significant difference that could indicate the effects on height are nonadditive, though more data from extremely tall individuals would be necessary to provide definitive evidence.

We also tested whether height scores were correlated with actual height in 407 individuals from the ADNI and Cache County datasets for each individual with both height and genetic data available. We tested for a correlation between the two using Pearson's product moment correlation coefficient, which is calculated using the R statistical package [26].

## 3. Results and Discussion

We tested whether a simple polygenic height score, calculated using SNPs that were statistically associated with human height in the GIANT consortium data [10], could accurately predict Mr. Bradley's height rank amongst 1020 individuals of Northern European descent. We used Haploview to identify tag SNPs for each LD block across the 22,539 GIANT SNPs to avoid counting a single signal multiple times and to estimate how many independent signals exist in the GIANT SNPs. Using the Alzheimer's Disease Genetics Consortium (ADGC) [22, 23] data with over 30,000 individuals, we identified 3428 unique signals, suggesting that most of the GIANT SNPs tag redundant effects. This is consistent with the GIANT result that most of the adult height variability explained by their SNPs is captured in the top 697 SNPs identified. After extracting genome-wide significant GIANT SNPs from Mr. Bradley's exome and SNP data and using only a single tag SNP within each linkage disequilibrium (LD) block, 2910 SNPs (2491 genotyped, 419 imputed, Supplementary Table 1) remained and were included in the analysis. These represent 2910 of the 3428 LD blocks identified across the 22,539 significant GIANT SNPs using the ADGC dataset. Each allele included in this study is estimated by the GIANT consortium to affect an individual's height by −0.14 to 0.19 millimeters.

**Figure 2:**
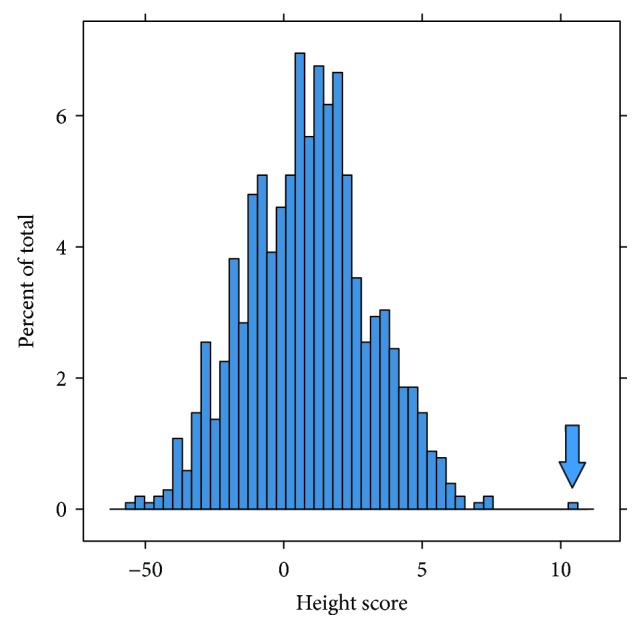
Height score distribution calculated using the 2910 SNPs. Mr. Bradley's height score (10.32, indicated by the arrow) ranked highest when compared to the 1020 individuals from ADNI and Cache County, while the next highest was 7.43. The mean height score within the ADNI and Cache County data was 0.98 with a standard deviation of 2.22, making Mr. Bradley's height score 4.2 standard deviations above the mean.

We calculated height scores weighted by effect size (see Supplementary Table 1 for effect betas) for Mr. Bradley and 1020 individuals from the Alzheimer's Disease Neuroimaging Initiative (ADNI) and the Cache County Study on Memory Health and Aging. Because Mr. Bradley's height is 8.6 standard deviations above the average height of a male in the US, it is expected that his height score would be much higher than the average of the 1020 individuals for whom height scores were calculated. Mr. Bradley's height score (10.32), calculated using the 2910 SNPs, was ranked highest, while the next highest was 7.43 (Figure 2). The mean height score within the ADNI and Cache County data was 0.98 with a standard deviation of 2.22, making Mr. Bradley's height score 4.2 standard deviations above the mean, as expected.

In order to determine how few SNPs could be used for Mr. Bradley's height score to rank highest when compared to the ADNI and Cache County population data, we created subsets of SNPs randomly from the 2910 available SNPs and then calculated height scores for all 1020 individuals as well as Mr. Bradley. We then ranked the resulting height scores and recorded Mr. Bradley's percentile (Table 1). This procedure was replicated 1 million times for each SNP subset size. Choosing a subset of 100 SNPs randomly 1 million times, Mr. Bradley's height scores calculated from the SNP subsets range from the lowest to the highest when compared to the ADNI and Cache County SNP subset height scores. His median height percentile settles at 96.9. Using a subset of 250 SNPs across 1 million iterations, Mr. Bradley's median height percentile rises to 99.6 with his minimum height percentile at 20.4 and his maximum ranking highest. By using 750 SNPs, Mr. Bradley's Q1 height rank is the top of the distribution, meaning that at least 75% of the time, his height score was ranked highest in the distribution. His lowest percentile using 750 SNPs was 78.8. Randomly selecting 1500 of the 2910 SNPs, Mr. Bradley's lowest rank was in the 99.2 percentile (1017 of 1021).

We also explored the difference in height scores between the observed distribution of height scores amongst the 1020 individuals from ADNI and Cache County when compared to the null distribution, based on 20 billion simulated individuals created from ADNI and Cache County genotypes, assuming no evolutionary constraints. The mean simulated height score (−0.30) was 1.28 mm lower than the observed height score mean (0.98). The maximum simulated height score (8.37) was 1.95 mm lower than Mr. Bradley's (10.32).

We tested whether Mr. Bradley's extreme height may be caused by an increased proportion of heterozygous or homozygous genotypes using a goodness-of-fit test (*p* = 1.28 × 10^−24^). Mr. Bradley has an increased proportion of homozygous genotypes for alleles with a positive effect (Table 2). He has nearly identical numbers of heterozygous genotypes for positive (associated with increased height) and negative (associated with decreased height) effect sizes with 621 and 634, respectively. The additive effects on his score for the positive and negative heterozygous genotypes are approximately equal and opposite at 15.12 and −15.27, respectively, summing to −0.17. There is a large difference, however, when comparing the homozygous genotypes for alleles with a positive and negative effect. Mr. Bradley has 465 genotypes where he is homozygous for GIANT alleles with a positive effect and only 267 genotypes where he is homozygous for GIANT alleles with a negative effect. The additive effects where Mr. Bradley is homozygous for positive and negative alleles are 25.89 and −15.42, respectively. The sum of all four scores equates to his height score of 10.32. Based on these data, Mr. Bradley's height score rank is largely attributed to an excess of 198 positive-effect homozygous genotypes.

**Figure 3:**
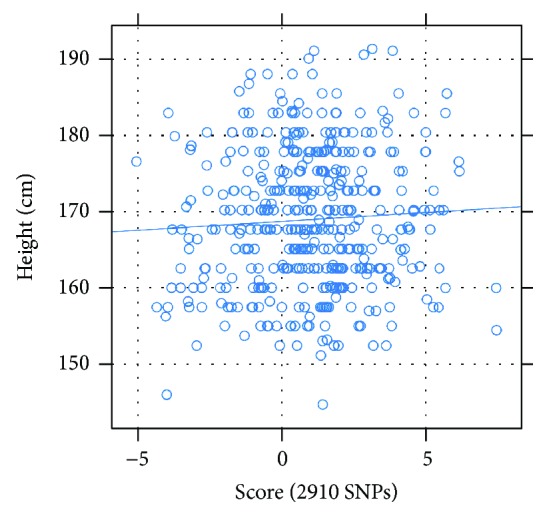
Correlation between height scores and self-reported height in the ADNI and Cache County individuals. We plotted height scores and self-reported heights (at age 18) for individuals in the ADNI and Cache County datasets and found poor correlation between the two. We also calculated the Pearson product moment correlation coefficient (correlation coefficient = 0.06, *p* = 0.25).

Using available height data from the ADNI and Cache County data, we tested whether the height scores calculated using the 2910 SNPs were correlated with the self-reported heights (at age 18) for the 407 individuals for which we have both height and genetic data. We failed to detect significant correlation between the two (correlation coefficient = 0.06, *p* = 0.25; Figure 3). This is consistent with the findings of the GIANT consortium. With a population of 1914 individuals, Wood et al. found a predictive *r* ^2^ = 0.14 for 697 SNPs (20% variation explained) [10]. It is expected that this *r* ^2^ should be stronger than the correlation coefficient in our findings because of our smaller population size of 407 individuals of the ADNI and Cache County individuals as well as the fact that the GIANT consortium identified the 697 SNPs used for prediction directly from their population of 1914 individuals.

## 4. Conclusions

While research has shown that height is a polygenic trait heavily influenced by common SNPs [7–12], a polygenic score that quantifies common SNP effect is generally insufficient for successful individual phenotype prediction. We demonstrate that in the case of Mr. Bradley, a rare combination of common SNPs corresponds to an extremely high polygenic score that predicts an extreme phenotype. Because Mr. Bradley is an outlier, studying his genetic makeup provides a unique context to understand the complex nature of human height. Using a simple polygenic model across approximately 2000 SNPs, we accurately predicted Mr. Bradley's height rank amongst a population of 1020 individuals.

The accurate prediction of tall individuals based on polygenic score has been found by both Chan et al. [13] and Liu et al. [14], confirming that in the case of an extremely tall phenotype, such as Mr. Bradley's, polygenic scores can predict height rank. While these studies used a population of tall individuals to confirm their findings, we provide a validation of one individual polygenic height score rather than a distribution.

Mr. Bradley's height score—like his actual height—was an extreme outlier (4.2 standard deviations above the mean). This appears to be driven by an increased proportion of homozygous genotypes for SNPs associated with increased height when compared to the average ADNI and Cache County genotype values. Despite this, his height score only predicted him to be 10.32 mm taller than average. This suggests that while Mr. Bradley's extreme polygenic score could accurately rank his height amongst 1020 individuals, it does not accurately predict his actual height measurement, demonstrating that there are significant factors unaccounted for. Similarly, and as expected, this model was not able to accurately predict actual heights among the 407 ADNI and Cache County individuals for which we had both height and genetic data. These results as well as Mr. Bradley's predicted height (10.32 mm taller than average) suggest that other factors such as environmental factors [27], nonadditive individual loci [28], and both epistasis (gene by gene interactions) and gene by environment interactions [29] play a significant role in determining actual height measurement. Recent studies of heritability in height and other complex traits suggest significant contributions of nonadditive factors [30, 31].

Height is a complex trait that may serve as an effective phenotype model for other complex traits and diseases because it is a noninvasive and easily-measured phenotype to study. By developing new models and studies to better understand all genetic contributors to an individual's height, researchers will be able to apply the methods to other complex data.

## Figures and Tables

**Table 1:** Mr. Bradley's height score percentiles when compared to the population data for random subsets of SNPs.

Set size	100	250	500	750	1000	1250	1500	1750	2000
Min	0	20.4	54.4	78.8	94.3	97.1	99.2	99.6	99.6
Q1	89.6	98.2	99.8	^∗^	^∗^	^∗^	^∗^	^∗^	^∗^
Median	96.9	99.6	^∗^	^∗^	^∗^	^∗^	^∗^	^∗^	^∗^
Q3	99.3	99.9	^∗^	^∗^	^∗^	^∗^	^∗^	^∗^	^∗^
Max	^∗^	^∗^	^∗^	^∗^	^∗^	^∗^	^∗^	^∗^	^∗^

Table 1 Shawn Bradley's height score quickly stabilizes at the highest rank as SNP-set size increases. Data are represented in percentiles. The “∗” indicates that his score was the highest.

**Table 2:** Genotype counts for effect alleles in Shawn Bradley and the ADNI/Cache County populations.

	Shawn Bradley			Average across ADNI/Cache County		
	Homozygous for effect allele (additive effect on score)	Heterozygous (additive effect on score)	Homozygous for noneffect allele	Homozygous for effect allele	Heterozygous	Homozygous for noneffect allele
Positive effect	465 (25.89)	621 (15.12)	347 (NA)	428 (22.72)	552 (13.01)	479 (NA)
Negative effect	267 (−15.42)	634 (−15.27)	510 (NA)	416 (−22.28)	535 (−12.60)	497 (NA)

## Data Availability

The data used to support the findings of this study are included within the article.

## Supplementary Materials

Supplementary Materials Supplementary Table 1: provides the 2910 SNPs used to create the height scores as well as the beta scores from the GIANT consortium for each SNP.
